# Early Description of Common Embryonic Origin of Skin and Nervous System by Avicenna

**DOI:** 10.31661/gmj.v13i.3330

**Published:** 2024-03-09

**Authors:** Sobhan Ghezloo, Babak Daneshfard, Ebrahim Khadem

**Affiliations:** ^1^ Department of History of Medicine, School of Persian Medicine, Tehran University of Medical Science, Tehran, Iran; ^2^ Chronic Respiratory Diseases Research Center, National Research Institute of Tuberculosis and Lung Diseases, Shahid Beheshti University of Medical Sciences, Tehran, Iran; ^3^ Persian Medicine Network, Universal Scientific Education and Research Network, Tehran, Iran; ^4^ Department of Persian Medicine, School of Persian Medicine, Tehran University of Medical Sciences, Tehran, Iran

**Keywords:** Avicenna, Embryology, Histology, History of Medicine, Persian Medicine

## Dear Editor,

Undoubtedly, today’s advances in medical science in all branches are the result of the efforts and achievements of scientists of various Western and Eastern nations, which began a long time ago and have continued until today.

Persian Medicine (PM), is one of the ancient medical schools that is based on the theory of four humors while its history goes back to the 7th century BC [1]. Persian Medicine physicians have recognized and defined the function of different body parts [2]; many of their findings have been verified today. They were relying on their experiences while they had no access to our modern facilities [3].

Abu Ali al-Hussein ibn Abdullah ibn Sina (Avicenna) was born around 980 AD in a city near Bukhara. When he reached the age of ten, he learned law and mathematics. Then, he studied with Hakim Aba Abdullah al-Natili. It was during this period that he gradually developed an interest in medicine and it is said that Abu Mansur al-Hasan ibn Nuh al-Qumri was one of Ibn Sina’s teachers in medicine. At the age of 22, Avicenna was considered the most famous physician of his time [4].

He wrote most of his books including The Canon of Medicine in Arabic (as the Lingua Francas of that era), although he has works in Persian (such as Daneshnameh-ye Alai) which was his mother language. The Canon was translated and edited 15 times in Hebrew and Latin languages. For almost 600 years, it was a fundamental medical literature in schools in the East, as well as Western universities [5].

The skin is the largest single organ of the body which has an important role in the immune system [6]. It has a significant importance in view of Persian Medicine physicians such as Avicenna and Rhazes.

According to Iranian physicians, the skin is a nervous tissue, and Persian Medicine physicians have talked about the similarity of the embryonic origin of skin and nerves [7][8]. Persian Medicine sages were aware of the importance of the skin as a defense barrier to the body. Along with the resistance and strength of the skin in protecting the internal organs, they considered the pores in the skin as a way to expel the waste materials. The presence of sensory nerves in the skin for quick understanding and quick response to environmental stimuli is another feature that Iranian sages had considered for the skin [7].

In different parts of Canon, Avicenna mentions the nervous origin of the skin. In the first book of Canon, he clearly emphasizes that: "The origin of the nerves, as it is well known, is the brain. The most extreme of their division is the skin" [9].

In the continuation of this chapter of Canon, he has described the path of different neural circuits in various organs in detail. He has also named the pair of nerves that lead to the skin of each specific organ [9].

Notably, it is well-known in modern-day embryology that the origin of both of the nervous system and skin is the ectoderm. During the 3rd to 8th weeks of the embryonic period, the ectodermal germ layer is induced to form the neural plate and then the neuroectoderm. The neural plate can be induced by upregulating fibroblast growth factor (FGF) signaling while inhibiting the activity of bone morphogenetic protein 4 (BMP4), which is a member of the transforming growth factor-B (TGF-B) family responsible for ventralizing ectoderm and mesoderm. On the other hand, the presence of BMP4, which permeates the mesoderm and ectoderm of the gastrulating embryo, induces the formation of epidermis in the ectoderm [10]. Avicenna’s attention to the delicacy of the skin structure and his precision in discovering the smallest parts of the skin is indeed surprising. Many features and details that Avicenna has mentioned about the structure of neural circuits in the skin have been shown today with advanced medical and laboratory equipment.

These innovations of Avicenna in describing the structure of the skin confirm his knowledge about its origin. Of course, there are many other points that require more in-depth investigations.

## Conflict of Interest

There is no conflict of interest to disclose.
